# Waning and boosting: on the dynamics of immune status

**DOI:** 10.1007/s00285-018-1239-5

**Published:** 2018-05-15

**Authors:** O. Diekmann, W. F. de Graaf, M. E. E. Kretzschmar, P. F. M. Teunis

**Affiliations:** 1 Department of Mathematics, Utrecht University, Utrecht, The Netherlands; 2 Julius Center for Health Sciences and Primary Care, University Medical Center Utrecht, Utrecht, The Netherlands; 3 Center for Infectious Disease Control, National Institute for Public Health and the Environment, Bilthoven, The Netherlands; 4 Hubert Department of Global Health, Rollins School of Public Health, Emory University, Atlanta, GA USA

**Keywords:** Waning of immunity, Boosting of immunity, Renewal equation, Stable distribution, Next-generation operator, 92D30, 60J75, 60K20

## Abstract

The aim is to describe the distribution of immune status (as captured by antibody level) on the basis of a within-host submodel for continuous waning and occasional boosting. Inspired by Feller’s fundamental work and the more recent delay equation formulation of models for the dynamics of physiologically structured populations, we derive, for given force of infection, a linear renewal equation. The solution is obtained by generation expansion, with the generation number corresponding to the number of times the individual became infected. Our main result provides a precise characterization of the stable distribution of immune status.

## Introduction

The immune system defends an individual host against pathogens. After infection with a specific species of pathogen has been cleared, some memory remains, providing protection against future attacks of that same pathogen. Over time the memory wanes, until it is boosted by a new encounter. Thus the immune status is shaped by a combination of the exogenous process of exposure and endogenous processes (fighting the invader to achieve clearance of the infection and subsequent waning).

In practice the immune status of an individual is often quantified by measuring the concentration of specific antibodies in serum. Distributions of such serological measurements are used to assess the immune status of a population, for example in the context of vaccination programs (Wilson et al. 2012). The immune status of a population impacts the risk of outbreaks of an infection and can provide information on incidence of infection, including asymptomatic infection (Metcalf et al. 2016). Longitudinal changes of antibody titers of individuals may show the effects of boosting and waning over time, e.g., for pertussis (Versteegh et al. 2005). In mathematical models for vaccine preventable diseases, immunity is often represented by a dichotomous variable -individuals are either susceptible or immune- even though this distinction is not straightforward in reality. Therefore, it is useful to have a mathematical modeling framework that is capable of describing immunity as a continuous variable subject to waning and boosting over time. Our aim here is to provide a first step towards such a framework. We neglect all subtleties of specific infectious diseases and focus on the processes of waning and boosting in their simplest form.

So we ignore much of the subtlety and complexity of the immune system by postulating that the immune status is fully described by a positive quantity *y* (antibody titer against pertussis toxin is what we have in mind as a concrete example). Waning is described by the ordinary differential equation $\mathrm {d}y/\mathrm {d}t = g(y)$ for the decline of *y* between encounters with the pathogen. Such encounters occur at rate $\Lambda$. So $\Lambda$ is the constant force of infection and is considered a parameter (in Sect. 5 we shall briefly indicate how to formulate a feedback consistency condition for $\Lambda$; this condition involves assumptions about infectiousness and thus increases the number of parameters). We assume that, on the time scale set by *g* and $\Lambda$, the time it takes the immune system to clear infection is negligible. This assumption allows us to introduce as a third model ingredient the instantaneous boosting map *f* that sends the immune status *y* just before the infection, to the immune status *f*(*y*) just after clearance of infection. In De Graaf et al. (2014) an explicit expression for *f* was derived from a submodel for the struggle between the pathogen and the immune system; see (Teunis et al. 2016) for a follow-up.

The three ingredients *g*, $\Lambda$ and *f* define a Piecewise Deterministic Markov Process (Davis 1993; Rudnicki and Tyran-Kamińska 2015). Indeed, waning and boosting are both deterministic, the only randomness is in the hitting times of the Poisson process with rate $\Lambda$. Let the random variable *Y*(*a*), with $Y(0)=y_b$, correspond to the immune status at age *a* of an immortal individual. Here $y_b$, the immune status at birth, is another parameter (heterogeneity can of course be captured by an assumed distribution of $y_b$). Provided the pathogen under consideration does not contribute to mortality, we can later on introduce an independent age-specific survival probability.

Having established this mathematical framework, we now want to answer the following questions:

- Can we compute the distribution of an individual’s immune status at age *a*, given that it starts life with an immune level $y_b$?
- If we let the process run for a long time, will the distribution of immune status converge to a stable distribution? Since feedback through $\Lambda$ on the transmission process is ignored, the stable distribution will describe the distribution of immunity in a population in steady state, if everybody is born with immune status $y_b$.

Let *y* be an element of $(0,\infty )$ and let $\Gamma$ be a measurable subset of $(0,\infty )$. Development over time of the distribution of immune status is described by the kernel

1.1 $$\begin{aligned} Q_t(y,\Gamma )={\mathbb {P}}(Y(a+t) \in \Gamma | Y(a) = y) \end{aligned}$$

which, by assumption, does not depend on *a*. In Sect. 2 we formulate a Renewal Equation (RE) for *Q* and solve it by generation expansion (using the techniques of Sect. 4 of Diekmann et al. (1998) one can show that *Q* does have, as it should, the Chapman–Kolmogorov property; in the “Appendix A1” we formulate the more traditional Kolmogorov backward and forward PDE that are associated with *Q*). In Sect. 3 we introduce the corresponding next-generation operator and construct its fixed point that describes the stable distribution at the generation level. A general result from (Diekmann et al. 1998) relates the stable distribution at the generation level to the stable distribution of the process itself. This result is formulated in Sect. 4.

**Fig. 1:**
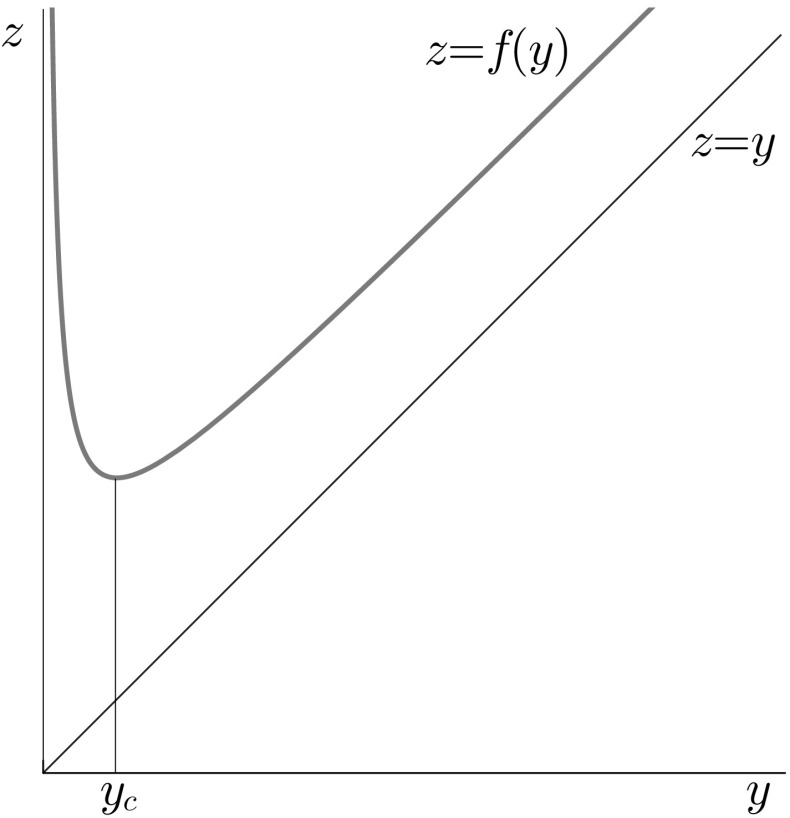
The graph of *f*

## The RE and its solution by generation expansion

Concerning the three model ingredients, *g*, $\Lambda$ and *f* we assume the following

$$H_{\Lambda }$$:
$\Lambda >0$, to ensure that exposure actually occurs.
$$H_{f}$$:
$f:(0,\infty )\rightarrow (0,\infty )$ is continuously differentiable and there exists $y_c\in (0,\infty )$ such that $f'(y)<0$ for $0<y<y_c$, $f'(y_c)=0$, $f'(y)>0$ for $y_c<y<\infty$. In addition, $\displaystyle \lim _{y\downarrow 0} f(y)=\infty$, $f(y)>y$ on $(0,\infty )$ and for some $\delta \ge 0$
  $$\begin{aligned} f(y)=y+\delta +o(1)\quad \text {for}\quad y\rightarrow \infty \end{aligned}$$
  Moreover, $f'(y)=1+o(1)$ for $y\rightarrow \infty$. See Fig. 1. See “Appendix A2” for an alternative. This form of *f*(*y*) is motivated by De Graaf et al. (2014) and implies that for an exposure occurring in an individual with immune status $y<y_c$ a large jump of immune level occurs during infection, while the increase in immune level is small if the immune status is higher than $y_c$ at exposure. We interpret the threshold $y_c$ as the immune level that distinguishes symptomatic and asymptomatic infection. In other words, an immune level $y>y_c$ provides protection against symptoms but nevertheless the encounter with the pathogen leads to a slight increase in immune level, whilst $y<y_c$ does not provide much protection and leads to a large boost of the immune level.
$$H_{g}$$:
The function *g* describes the rate of waning of immunity between exposures and should therefore ensure that *y*(*a*) is a monotone decreasing (but positive) function. So let $g:(0,\infty )\rightarrow (-\infty ,0)$ be such that the initial value problem
  $$\begin{aligned} \frac{\mathrm {d}y}{\mathrm {d}a}=g(y),\quad y(a_0)=y_0>0 \end{aligned}$$
  has a unique solution
  2.1 $$\begin{aligned} y(a)=\pi (a-a_0,y_0),\quad -\infty<a<\infty \end{aligned}$$
  and $$\lim _{a\rightarrow \infty } y(a)=0$$, $$\lim _{a\rightarrow -\infty } y(a)=\infty $$.

An alternative formulation of this assumption is that $\frac{1}{g}$ has a primitive *T*, say

2.2 $$\begin{aligned} T(y):=\int _{y_c}^y\frac{\mathrm {d}\eta }{g(\eta )} \end{aligned}$$

such that $T(y)\rightarrow \infty$ for $y\downarrow 0$ and $T(y)\rightarrow -\infty$ for $y\rightarrow \infty$. The relationship between $\pi$ and *T* is given by

2.3 $$\begin{aligned} \pi (a-a_0,y_0)=T^{-1}(a-a_0+T(y_0)) \end{aligned}$$

For later use we observe that

2.4 $$\begin{aligned} \frac{\partial \pi }{\partial y}(t,y)=\frac{\partial \pi }{\partial t}(t,y)\frac{1}{g(y)}=\frac{g(\pi (t,y))}{g(y)} \end{aligned}$$

An example is provided by

2.5 $$\begin{aligned} g(y)=-wy \end{aligned}$$

with $w>0$ a parameter. This choice for *g*(*y*) models an exponential decline in immune level between boosting events. In that case

2.6 $$\begin{aligned} \pi (a,y_0)=\mathrm {e}^{-wa}y_0 \end{aligned}$$

and

2.7 $$\begin{aligned} T(y)=\frac{1}{w}\log \left( \frac{y_c}{y}\right) ,\quad T^{-1}(a)=\mathrm {e}^{-wa}y_c \end{aligned}$$

In the generation expansion, we distinguish according to the number of hits of the Poisson process. So we start with the possibility of no hit at all.

### Definition 2.1

2.8 $$\begin{aligned} Q_t^0(y,\Gamma ):= & {} \mathrm {e}^{-\Lambda t}\delta _{\pi (t,y)}(\Gamma ) \nonumber \\= & {} {\mathbb {P}}\,(\text {no infection in } \, [a,a+t] \, \mathbf{and } \, Y(a+t)\in \Gamma |Y(a)=y)\qquad \end{aligned}$$

So $Q_t^0(y,\Gamma )$ describes the probability that an individual who has immune level *y* at age *a* and survives up to age $a+t$ has had no exposures in the time interval $[a,a+t]$ and has now an immune level in the set $\Gamma$ (e.g. within a given range $[y_{\text {low}},y_{\text {high}}]$). Of course, in this case we know exactly what the individual’s immune level is, but as soon as an exposure does occur, the immune level at $a+t$ has a range of possible values depending on when exactly the exposure occurred. Therefore, we now formulate an equation for the probability $Q_t(y,\Gamma )$, which is the probability that the individual has an immune state *y* in the set $\Gamma$ at age $a+t$ given that it had immune status *y* at age *a* and without any restriction on the number of exposures since then.

If an infection does occur in $[a,a+t]$, there has to be a first infection in this time window. The probability per unit of time that it occurs after exactly time $\sigma$ equals $\Lambda \mathrm {e}^{-\Lambda \sigma }$. In that case, the immune status jumps to $f(\pi (\sigma ,y))$ and there is time $t-\sigma$ left before the clock reaches *t*. Accordingly *Q* should satisfy the RE

2.9 $$\begin{aligned} Q_t(y,\Gamma )=Q_t^0(y,\Gamma )+\int _0^t\Lambda \mathrm {e}^{-\Lambda \sigma }Q_{t-\sigma }(f(\pi (\sigma ,y)),\Gamma )\mathrm {d}\sigma \end{aligned}$$

In order to rewrite (2.9) in a more condensed symbolic form we introduce another kernel. The kernel $B_t^1(y,\Gamma )$ describes the “position” on the *y*-axis immediately after the first jump (infection). Since it may happen that no jump occurs in the time interval of length *t* under consideration, this is not described by a probability distribution, but by a measure of total size less than one (this is sometimes called a “defective probability distribution”). The precise definition reads

### Definition 2.2

2.10 $$\begin{aligned} B_t^1(y,\Gamma ):= & {} \int _0^t\Lambda \mathrm {e}^{-\Lambda \sigma }\delta _{f(\pi (\sigma ,y))}(\Gamma )\mathrm {d}\sigma \nonumber \\= & {} {\mathbb {P}}\left( \text {infection does occur in } \, [a,a+t] \, \mathbf{and } \, Y \text { immediately}\right. \nonumber \\&\left. \text {after the first infection belongs to } \, \Gamma |Y(a)=y\right) \end{aligned}$$

In addition we define the **product** of two kernels as follows

### Definition 2.3

2.11 $$\begin{aligned} (\Phi \otimes \Psi )_t(y,\Gamma ):=\int _{[0,t]\times (0,\infty )}\Phi _{\mathrm {d}s}(y,\mathrm {d}z)\Psi _{t-s}(z,\Gamma ) \end{aligned}$$

These definitions allow us to write (2.9) as

2.12 $$\begin{aligned} Q=Q^0+B^1\otimes Q \end{aligned}$$

Successive approximation amounts to substituting the right hand side for *Q* at the right hand side, yielding

2.13 $$\begin{aligned} Q=Q^0+B^1\otimes Q^0+B^1\otimes B^1\otimes Q \end{aligned}$$

and then repeat this procedure again and again. By induction we define kernels $B^k$ for $k\ge 2$:

2.14 $$\begin{aligned} B^{k+1}:=B^1\otimes B^k,\quad k\ge 1 \end{aligned}$$

Explicitly we have

2.15 $$\begin{aligned} B_t^2(y,\Gamma )=\Lambda ^2\int _0^t\int _0^{t-\sigma _1}\mathrm {e}^{-(\sigma _1+\sigma _2)\Lambda }\delta _{f(\pi (\sigma _2,f(\pi (\sigma _1,y))))}(\Gamma )\mathrm {d}\sigma _2\mathrm {d}\sigma _1 \end{aligned}$$

and in general

2.16 $$\begin{aligned} B_t^k(y,\Gamma )=\Lambda ^k\int _{\Omega _{k{,}t}}\mathrm {e}^{-|\sigma |\Lambda }\delta _{F_k(t,\sigma ,y)}(\Gamma )\mathrm {d}\sigma \end{aligned}$$

with

2.17 $$\begin{aligned}&\Omega _{k{,}t}=\{\sigma \in {\mathbb {R}}^k:0\le \sigma _i\le t-\Sigma _{j=1}^{i-1}\sigma _j,\ i=1,2\ldots ,k\}\nonumber \\&|\sigma |:=\sigma _1+\sigma _2+\cdots +\sigma _k\nonumber \\&F_1(t,\sigma _1,y):=f(\pi (\sigma _1,y)),\quad 0\le \sigma _1\le t \nonumber \\&F_k(t,\sigma ,y):=F_{k-1}(t-\sigma _1,T_k\sigma , f(\pi (\sigma _1,y)))\nonumber \\&T_k\left( \! \begin{array}{c} \sigma _1 \\ \vdots \\ \sigma _k \end{array} \!\right) =\left( \! \begin{array}{c} \sigma _2 \\ \vdots \\ \sigma _k \end{array} \!\right) \end{aligned}$$

The interpretation reads

$$\begin{aligned} B_t^k(y,\Gamma )= & {} {\mathbb {P}}\left( \text {at least}\, k \, \text {infections do occur in} \, [a,a+t] \, \mathbf{and } \, Y \, \text {immediately}\right. \nonumber \\&\left. \text {after the } \, k\text {-th infection belongs to } \, \Gamma |Y(a)=y\right) \end{aligned}$$

The formulas (2.15) and (2.16) clearly show that all randomness derives from the hitting times of the Poisson process.

Returning to (2.13) and its “successors” obtained by repeating the approximation procedure, we are led to introduce, for $k\ge 1$

2.18 $$\begin{aligned} Q^k:=B^k\otimes Q^0 \end{aligned}$$

and to observe that

$$\begin{aligned} Q_t^k(y,\Gamma )= & {} {\mathbb {P}}\left( \text {exactly} \, k \, \text { infections in } \, [a,a+t] \, \mathbf{and } \, Y(a+t)\in \Gamma |Y(a)=y\right) \end{aligned}$$

The upshot is that we define

2.19 $$\begin{aligned} Q=\sum _{k=0}^{\infty }Q^k \end{aligned}$$

Cautionary note on notation: *Q* differs from $Q^1$.

Thus we constructed $Q_a(y_b,\cdot )$, the distribution of the random variable *Y*(*a*), given its value $y_b$ at birth, on the basis of the three model ingredients $\pi$, $\Lambda$ and *f*.

## The next-generation operator and its fixed point

In this section we define an operator, which maps the distribution of immune status right after an infection event to the distribution right after the next such event. We call this the next-generation operator and we will show that, under certain conditions, this operator has a unique fixed point (and that the fixed point has a density). In the next section we shall use these results to derive the existence and uniqueness of a stationary distribution of immunity to which, under the influence of repeated episodes of waning interrupted by boosting events, a general distribution converges for *t* tending to infinity.

If we consider the limit $t\rightarrow \infty$ for the kernel $B_t$, the probability that no infection occurs vanishes. So

3.1 $$\begin{aligned} B_{\infty }^1(y,\cdot ):=\int _0^{\infty }\Lambda \mathrm {e}^{-\Lambda \sigma }\delta _{f(\pi (\sigma ,y))}(\cdot )\mathrm {d}\sigma \end{aligned}$$

describes the probability distribution immediately after the next infection, given that the current immune status equals *y*, when we do not restrict the length of the time interval under consideration. Note that indeed for all *y* we have $B_{\infty }^1(y,(0,\infty ))=1$, reflecting what was stated above: in an infinite time interval infection occurs with probability 1. With this kernel we associate the map *K* defined by

3.2 $$\begin{aligned} (Kb)(\Gamma ):=\int _{(0,\infty )}B_{\infty }^1(\eta ,\Gamma )b(\mathrm {d}\eta ) \end{aligned}$$

The measure *b* describes the probability distribution of immune status of an individual directly after an infection event. The support of *b* is contained in the range of *f*, i.e. in $[f(y_c),\infty )$, since infection results in a new immune status obtained by applying *f* to the old immune status. Note that *b* is a probability measure, i.e., is positive and has total measure one. The map *K* preserves these properties. We call *K* the next-generation operator. Our aim in this section is to derive conditions on *f* and *g* that guarantee that *K* has a unique fixed point.

In order to derive a more handsome representation of *K*, we need some definitions. Since for every point in the range of *f* there are two points in the domain that are mapped to it, the inverse of *f* is double valued and we need notation to distinguish these two values from each other.

### Definition 3.1

3.3 $$\begin{aligned}&f_{-}^{-1}\ \text {is the inverse of } \, f \text { taking values less than } y_c \nonumber \\&f_+^{-1}\ \text {is the inverse of } \, f \text { taking values bigger than } y_c \end{aligned}$$

Both of these functions are defined on $[f(y_c),\infty )$. Both take the value $y_c$ in $f(y_c)$.

For $y_1>y_2$, let ${\hat{\tau }}(y_1,y_2)$ be the time involved in decreasing, by waning, from immune level $y_1$ to $y_2$. As it is convenient to have ${\hat{\tau }}$ also defined when $y_1>y_2$ does not hold, we extend by zero.

### Definition 3.2

3.4 $$\begin{aligned} {\hat{\tau }}(y_1,y_2):=\left\{ \begin{array}{lll} 0&{}\quad \mathrm {if}&{}y_2\ge y_1\\ \text {solution of}\ \pi (\tau ,y_1)=y_2&{}\quad \mathrm {if}&{}y_2\le y_1 \end{array} \right. \end{aligned}$$

Hence [cf. (2.2) and (2.3)]

3.5 $$\begin{aligned} {\hat{\tau }}(y_1,y_2)=T(y_2)-T(y_1)\quad \text {if}\quad y_2\le y_1 \end{aligned}$$

We also introduce the notation

3.6 $$\begin{aligned} \Gamma _y:=[f(y_c),y) \end{aligned}$$

The motivation for our special interest in sets of this form is the following. Measures in the range of *K* have their support in $[f(y_c),\infty )$. As our interest is in iterating *K*, we might as well restrict the domain of *K* to probability measures concentrated on $[f(y_c),\infty )$. The values that such measures take on sets of the form (3.6), for arbitrary $y>0$, provide full information about the measure.

Next note that

3.7 $$\begin{aligned} (Kb)(\Gamma _y)=\iint _{\Delta (y)}\Lambda \mathrm {e}^{-\Lambda \sigma }b(\mathrm {d}\eta )\mathrm {d}\sigma \end{aligned}$$

with

3.8 $$\begin{aligned} \Delta (y)=\{(\sigma ,\eta ):\sigma \ge 0,\,\eta \ge f(y_c),\,f(\pi (\sigma ,\eta ))<y\}\end{aligned}$$

Since (recall Fig. 1 and Definitions 3.1 and 3.2)

$$\begin{aligned} \begin{aligned} f(\pi (\sigma ,\eta ))<y&\Longleftrightarrow \ f_{-}^{-1}(y)<\pi (\sigma ,\eta )<f_+^{-1}(y) \\&\Longleftrightarrow \ {\hat{\tau }}(\eta ,f_+^{-1}(y))<\sigma <{\hat{\tau }}(\eta ,f_{-}^{-1}(y)) \end{aligned} \end{aligned}$$

we can perform the integration with respect to $\sigma$ and rewrite (3.7) in the form

3.9 $$\begin{aligned} (Kb)(\Gamma _y)=\int _{[f(y_c),\infty )}\left[ \mathrm {e}^{-\Lambda {\hat{\tau }}(\eta ,f_+^{-1}(y))}-\mathrm {e}^{-\Lambda {\hat{\tau }}(\eta ,f_{-}^{-1}(y))}\right] b(\mathrm {d}\eta ) \end{aligned}$$

The first term at the right hand side limits starting points for jumps to the right of $y_c$, the second limits starting points to the left of $y_c$. As the distinction is helpful, we emphasize it by defining

3.10 $$\begin{aligned} K=K_++K_- \end{aligned}$$

with

3.11 $$\begin{aligned} (K_+b)(\Gamma _y):=\int _{[f(y_c),\infty )}\left[ \mathrm {e}^{-\Lambda {\hat{\tau }}(\eta ,f_+^{-1}(y))}-\mathrm {e}^{-\Lambda {\hat{\tau }}(\eta ,y_c)}\right] b(\mathrm {d}\eta ) \end{aligned}$$

and

3.12 $$\begin{aligned} (K_-b)(\Gamma _y):= & {} \int _{[f(y_c),\infty )} \left[ \mathrm {e}^{-\Lambda {\hat{\tau }}(\eta ,y_c)}- \mathrm {e}^{-\Lambda {\hat{\tau }}(\eta ,f_{-}^{-1}(y))} \right] b(\mathrm {d}\eta ) \nonumber \\= & {} c\left( 1-\mathrm {e}^{-\Lambda T(f_{-}^{-1}(y))}\right) \end{aligned}$$

with

3.13 $$\begin{aligned} c:=\int _{[f(y_c),\infty )}\mathrm {e}^{\Lambda T(\eta )}b(\mathrm {d}\eta ) \end{aligned}$$

Note that $T(\eta )<0$ for $\eta \ge f(y_c)$, cf. (2.2), so *c* is well-defined. The range of $K_-$ is one-dimensional and spanned by an absolutely continuous measure. This reflects that, in order to have immune status less than $y_c$ when the next infection hits, the immune status must first wane to $y_c$ without any hit (the probability that this happens is $\mathrm {e}^{\Lambda T(\eta )}$ and determines the contribution to *c*). But once $y_c$ is “safely” reached, any information about $\eta$ is irrelevant. After arrival in $y_c$, the waiting time till being hit is exponentially distributed with parameter $\Lambda$ and the waiting time translates to an arrival position after the infection, as detailed in (3.12).

Now, let us focus attention on $K_+$. For $y\le f^{(2)}(y_c):=f(f(y_c))$ we have $f_+^{-1}(y)\le f(y_c)$ and hence ${\hat{\tau }}(\eta ,f_+^{-1}(y))=T(f_+^{-1}(y))-T(\eta )$ for all $\eta \ge f(y_c)$. Consequently

3.14 $$\begin{aligned} (K_+b)(\Gamma _y)=c\left( \mathrm {e}^{-\Lambda T(f_{+}^{-1}(y))}-1\right) \quad \text {for}\quad y\le f^{(2)}(y_c) \end{aligned}$$

For $y> f^{(2)}(y_c)$, on the other hand, we have ${\hat{\tau }}(\eta ,f_+^{-1}(y))=0$ for $f(y_c)\le \eta \le f_+^{-1}(y)$ and consequently

3.15 $$\begin{aligned} (K_+b)(\Gamma _y)= & {} \int _{[f(y_c),f_{+}^{-1}(y))}b(\mathrm {d}\eta )+\mathrm {e}^{-\Lambda T(f_{+}^{-1}(y))} \nonumber \\&\times \int _{[f_{+}^{-1}(y),\infty )}\mathrm {e}^{\Lambda T(\eta )}b(\mathrm {d}\eta )-c\quad \text {for}\quad y> f^{(2)}(y_c) \end{aligned}$$

Combining (3.12) and (3.14) we see that a measure in the range of *K* has a density on $[f(y_c),f^{(2)}(y_c)]$. The reason is similar as given above concerning $K_-$: in order to lead to an arrival position in $[f(y_c),f^{(2)}(y_c)]$, the immune status has to wane to below $f(y_c)$ and information about $\eta$ becomes irrelevant upon passing $f(y_c)$. We can subsequently use the explicit representation (3.15) to conclude that a measure in the range of $K^2$ has a density on $[f(y_c),f^{(3)}(y_c)]$. Etcetera. It follows that a fixed point of *K* necessarily has a density.

Our aim is now to construct a fixed point of *K*. Apart from a multiplicative constant *c*, the fixed point is known when we restrict attention to the interval $[f(y_c),f^{(2)}(y_c)]$, cf. (3.12) and (3.14). The idea is to use (3.15) to extend the interval on which we know the fixed point and in the very end use the condition that the construction should yield a probability measure to determine the free constant *c*.

Assuming that *b* has a density $\phi$ we can, using (3.12) and (3.15), write the fixed point problem $Kb=b$ in the form

3.16 $$\begin{aligned} \int _{f(y_c)}^y\phi (\eta )\mathrm {d}\eta= & {} \int _{f(y_c)}^{f_{+}^{-1}(y)}\phi (\eta )\mathrm {d}\eta +\mathrm {e}^{-\Lambda T(f_{+}^{-1}(y))}\int _{f_{+}^{-1}(y)}^{\infty }\mathrm {e}^{\Lambda T(\eta )}\phi (\eta )\mathrm {d}\eta \nonumber \\&-\mathrm {e}^{-\Lambda T(f_-^{-1}(y))}c\quad \text {for}\quad y> f^{(2)}(y_c) \end{aligned}$$

and with $\phi$ for $f(y_c)\le y\le f^{(2)}(y_c)$ defined by

3.17 $$\begin{aligned} \phi (y)=\frac{\mathrm {d}}{\mathrm {d}y}\,c\left( \mathrm {e}^{-\Lambda T(f_{+}^{-1}(y))}-\mathrm {e}^{-\Lambda T(f_-^{-1}(y))}\right) \end{aligned}$$

The factor $\int _{f_{+}^{-1}(y)}^{\infty }\mathrm {e}^{\Lambda T(\eta )}\phi (\eta )\mathrm {d}\eta$ at the right hand side of (3.16) involves values of $\phi$ beyond *y* and this makes the use of (3.16) for extending the fixed point from the explicit (apart from an as yet unknown constant *c*) expressions provided by the right hand sides of (3.12) and (3.14) to larger values of *y* problematic. The solution is to differentiate (3.16) with respect to *y* and next combine the two identities to eliminate the factor. This is just a formula manipulation trick that has no biological interpretation whatsoever. We realize that unpleasant technical details are involved, but are not able to avoid these. To facilitate the formulation, we introduce some notation.

### Definition 3.3

3.18 $$\begin{aligned}&\alpha (y):=-\Lambda \frac{\mathrm {d}}{\mathrm {d}y}T(f_+^{-1}(y))=-\Lambda \frac{1}{g(f_+^{-1}(y))}\frac{1}{f'(f_+^{-1}(y))}>0 \end{aligned}$$

3.19 $$\begin{aligned}&\beta (y):=\Lambda \frac{\mathrm {d}}{\mathrm {d}y}T(f_-^{-1}(y))=\Lambda \frac{1}{g(f_-^{-1}(y))}\frac{1}{f'(f_-^{-1}(y))}>0 \end{aligned}$$

Differentation of (3.16) yields^1^

$$\begin{aligned} \phi (y)=\alpha (y)\mathrm {e}^{-\Lambda T(f_{+}^{-1}(y))}\int _{f_{+}^{-1}(y)}^{\infty }\mathrm {e}^{\Lambda T(\eta )}\phi (\eta )\mathrm {d}\eta +\beta (y)\mathrm {e}^{-\Lambda T(f_-^{-1}(y))}c \end{aligned}$$

which in combination with (3.16) itself leads to

3.20 $$\begin{aligned} \phi (y)=\alpha (y)\int _{f_{+}^{-1}(y)}^y\phi (\eta )\mathrm {d}\eta +(\alpha (y)+\beta (y))\mathrm {e}^{-\Lambda T(f_-^{-1}(y))}c \end{aligned}$$

Equation (3.20) should hold for $y>f^{(2)}(y_c)$ and is supplemented by (3.17), which we rewrite as

3.21 $$\begin{aligned} \phi (y)=\left( \alpha (y)\mathrm {e}^{-\Lambda T(f_+^{-1}(y))}+\beta (y)\mathrm {e}^{-\Lambda T(f_-^{-1}(y))}\right) c\quad \text {for}\quad f(y_c)\le y\le f^{(2)}(y_c)\nonumber \\ \end{aligned}$$

Before analysing (3.20), let us check that no information was lost when deriving (3.20) from (3.16).

### Lemma 3.4

Assume that $\phi$ is integrable over $[f(y_c),\infty )$, satisfies (3.20) for $y>f^{(2)}(y_c)$ and (3.21) for $y\in [f(y_c),f^{(2)}(y_c)]$. Then (3.16) holds.

### Proof

We write (3.20) in the form

$$\begin{aligned} \begin{aligned} \frac{\mathrm {d}}{\mathrm {d}y} \int _{f(y_c)}^y\phi (\eta )\mathrm {d}\eta&=\alpha (y)\left( \int _{f(y_c)}^y\phi (\eta )\mathrm {d}\eta -\int _{f(y_c)}^{f_+^{-1}(y)}\phi (\eta )\mathrm {d}\eta +\mathrm {e}^{-\Lambda T(f_-^{-1}(y))}c\right) \\&\quad -\frac{\mathrm {d}}{\mathrm {d}y}\mathrm {e}^{-\Lambda T(f_-^{-1}(y))}c \end{aligned} \end{aligned}$$

Now observe that for **any** integrable $\phi$ and for $y\ge f^{(2)}(y_c)$

i. $$\begin{aligned} \frac{\mathrm {d}}{\mathrm {d}y} \int _{f(y_c)}^{\infty }\mathrm {e}^{-\Lambda {\hat{\tau }}(\eta ,f_+^{-1}(y))}\phi (\eta )\mathrm {d}\eta =\alpha (y)\mathrm {e}^{-\Lambda T(f_+^{-1}(y))}\int _{f_+^{-1}(y)}^{\infty }\mathrm {e}^{\Lambda T(\eta )}\phi (\eta )\mathrm {d}\eta \end{aligned}$$
ii. $$\begin{aligned} \begin{aligned} \int _{f(y_c)}^{\infty }\mathrm {e}^{-\Lambda {\hat{\tau }}(\eta ,f_+^{-1}(y))}\phi (\eta )\mathrm {d}\eta&=\int _{f(y_c)}^{f_+^{-1}(y)}\phi (\eta )\mathrm {d}\eta \\&\quad +\mathrm {e}^{-\Lambda T(f_+^{-1}(y))}\int _{f_+^{-1}(y)}^{\infty }\mathrm {e}^{\Lambda T(\eta )}\phi (\eta )\mathrm {d}\eta \end{aligned} \end{aligned}$$

Combining these two identities we deduce

iii. $$\begin{aligned} \begin{aligned} \alpha (y)\int _{f(y_c)}^{f_+^{-1}(y)}\phi (\eta )\mathrm {d}\eta&=-\frac{\mathrm {d}}{\mathrm {d}y}\int _{f(y_c)}^{\infty } \mathrm {e}^{-\Lambda {\hat{\tau }}(\eta ,f_+^{-1}(y))} \phi (\eta )\mathrm {d}\eta \\&\quad +\alpha (y)\int _{f(y_c)}^{\infty } \mathrm {e}^{-\Lambda {\hat{\tau }} (\eta ,f_+^{-1}(y))}\phi (\eta )\mathrm {d}\eta \end{aligned} \end{aligned}$$

Identity iii. allows us to rewrite the rewritten (3.20) as

$$\begin{aligned}&\frac{\mathrm {d}}{\mathrm {d}y}\left[ \int _{f(y_c)}^y\phi (\eta )\mathrm {d}\eta -\int _{f(y_c)}^{\infty }\mathrm {e}^{-\Lambda {\hat{\tau }}(\eta ,f_+^{-1}(y))}\phi (\eta )\mathrm {d}\eta +\mathrm {e}^{-\Lambda T(f_-^{-1}(y))}c\right] \\&\quad =\alpha (y)\left[ \int _{f(y_c)}^y\phi (\eta )\mathrm {d}\eta -\int _{f(y_c)}^{\infty }\mathrm {e}^{-\Lambda {\hat{\tau }}(\eta ,f_+^{-1}(y))}\phi (\eta )\mathrm {d}\eta +\mathrm {e}^{-\Lambda T(f_-^{-1}(y))}c\right] \end{aligned}$$

Integrating the initial condition, see (3.17), we find that for $y=f^{(2)}(y_c)$ the quantity in the square brackets equals zero. By uniqueness, it equals zero for all $y\ge f^{(2)}(y_c)$. Using identity ii. above we see that this amounts to (3.16). $\square$

Standard contraction arguments (or, alternatively, monotone iteration arguments) guarantee that $\phi$ defined by (3.21) on $[f(y_c),f^{(2)}(y_c)]$ can be extended via (3.20) to an interval having $f^{(2)}(y_c)$ as its left end point. By continuation we obtain a maximal existence interval. Provided $\alpha$ is non-singular, this interval is $[f(y_c),\infty )$, since the equation is linear. A key point for us, however, is that $\phi$ should in fact be integrable over $[f(y_c),\infty )$. In other words, we want an a priori bound on the $L_1$-norm.

To derive some intuition, let us briefly look at the constant coefficient homogeneous version

3.22 $$\begin{aligned} \phi (y)=\alpha \int _{y-\delta }^{y}\phi (\eta )\mathrm {d}\eta \end{aligned}$$

This equation has a solution of the form $\phi (y)=\mathrm {e}^{\lambda y}$ provided $\lambda$ is a root of the characteristic equation

3.23 $$\begin{aligned} 1=\alpha \,\frac{1-\mathrm {e}^{-\lambda \delta }}{\lambda } \end{aligned}$$

(where the right hand side should be interpreted as $\alpha \delta$ for $\lambda =0$). Whenever $\alpha \delta <1$, all roots of (3.23) have negative real part (see e.g. Chapter XI of Diekmann et al. (1995)). If $\alpha \delta >1$, a positive real root exists. Clearly the corresponding solution is not integrable over the positive real axis.

This example illustrates that we need some kind of condition on the behaviour of $\alpha (y)$ and $f_+^{-1}(y)$ for $y\rightarrow \infty$. The probabilistic formulation is that we need tightness, meaning that mass is prevented from moving ever higher up the *y*-axis when we iterate *K*. The following consideration serves to build up intuition.

On average, the time in between successive jumps equals $\frac{1}{\Lambda }$. The condition

3.24 $$\begin{aligned} f(\pi (1/\Lambda ,y))<y\quad \text {for large } \, y \end{aligned}$$

states that high-up the *y*-axis we lose immunity if we jump after the expected time. As applying a nonlinear map and taking an expectation do not commute, this provides an idea, not a workable argument. Moreover, as we can also end up high on the *y*-axis by first waning to a very low *y*-level, we might need an additional and rather different condition to control this route to high immune levels. We now return to (3.20) and present sufficient conditions.

### Lemma 3.5

Assume that $\rho$ and *z* exist, with $0<\rho <1$ and $z\ge f^{(2)}(y_c)$, such that

3.25 $$\begin{aligned} f(\pi (\rho /\Lambda ,y))<y\quad \text {for}\quad y\ge f_+^{-1}(z) \end{aligned}$$

and

3.26 $$\begin{aligned} \int _z^y\alpha (\xi )\mathrm {e}^{-\Lambda T(f_-^{-1}(\xi ))}\mathrm {d}\xi \quad \text {is bounded for} \, y\in [z,\infty ) \end{aligned}$$

Then the unique solution of (3.20), (3.21) is integrable over $[f(y_c),\infty )$.

### Proof

Integrating (3.20) from *z* to $y>z$ we obtain

$$\begin{aligned} \int _z^y\phi (\xi )\mathrm {d}\xi =\int _z^y\alpha (\xi )\int _{f_{+}^{-1}(\xi )}^\xi \phi (\eta )\mathrm {d}\eta \mathrm {d}\xi +\int _z^y(\alpha (\xi )+\beta (\xi ))\mathrm {e}^{-\Lambda T(f_-^{-1}(\xi ))}c\,\mathrm {d}\xi \end{aligned}$$

Interchanging the two integrals in the first term at the right hand side leads to

$$\begin{aligned} \int _{f_{+}^{-1}(z)}^y\left( \int _{\text {max}\{z,\eta \}}^{\text {min}\{f(\eta ),y\}}\alpha (\xi )\mathrm {d}\xi \right) \phi (\eta )\mathrm {d}\eta \end{aligned}$$

Since $\alpha$ is positive and, moreover, a derivative (cf. (3.18))

$$\begin{aligned} \int _{\text {max}\{z,\eta \}}^{\text {min}\{f(\eta ),y\}}\alpha (\xi )\mathrm {d}\xi \le \int _{\eta }^{f(\eta )}\alpha (\xi )\mathrm {d}\xi =-\Lambda T(\eta )+\Lambda T(f_{+}^{-1}(\eta )) \end{aligned}$$

We claim that (3.25) implies that the right hand side is bounded by $\rho$. To verify this claim, we first write (3.25) as $\pi (\rho /\Lambda ,y)<f_{+}^{-1}(y)$ and next use (2.3) to reformulate this inequality as

$$\begin{aligned} T^{-1}\left( \frac{\rho }{\Lambda }+T(y)\right) <f_{+}^{-1}(y) \end{aligned}$$

Since *T* and $T^{-1}$ are decreasing, it follows that

$$\begin{aligned} \frac{\rho }{\Lambda }+T(y)>T(f_{+}^{-1}(y)) \end{aligned}$$

and, finally

$$\begin{aligned} -\Lambda T(y)+\Lambda T(f_{+}^{-1}(y))<\rho \end{aligned}$$

Returning to the identity at the start of the proof, we conclude that

$$\begin{aligned} \int _z^y\phi (\xi )\mathrm {d}\xi \le \rho \int _{f_{+}^{-1}(z)}^y\phi (\eta )\mathrm {d}\eta +\int _z^y(\alpha (\xi )+\beta (\xi ))\mathrm {e}^{-\Lambda T(f_-^{-1}(\xi ))}c\,\mathrm {d}\xi \end{aligned}$$

and hence

$$\begin{aligned} (1-\rho )\int _z^y\phi (\xi )\mathrm {d}\xi \le \rho \int _{f_{+}^{-1}(z)}^z\phi (\xi )\mathrm {d}\xi +\int _z^y(\alpha (\xi )+\beta (\xi ))\mathrm {e}^{-\Lambda T(f_-^{-1}(\xi ))}c\,\mathrm {d}\xi \end{aligned}$$

Now note that, on account of (3.19),

$$\begin{aligned} \begin{aligned} \int _z^y\beta (\xi )\mathrm {e}^{-\Lambda T(f_-^{-1}(\xi ))}\mathrm {d}\xi&=-\left. \mathrm {e}^{-\Lambda T(f_-^{-1}(\xi ))}\right| _z^y=\mathrm {e}^{-\Lambda T(f_-^{-1}(z))}-\mathrm {e}^{-\Lambda T(f_-^{-1}(y))} \\&\le \mathrm {e}^{-\Lambda T(f_-^{-1}(z))}\le 1 \end{aligned} \end{aligned}$$

since *T* is positive on $(0,y_c)$, cf. (2.2). Assumption (3.26) therefore guarantees that a constant *C* exists such that the right hand side, and hence the left hand side, is bounded by *C* for $y\in [z,\infty )$. It follows that $\int _z^y\phi (\xi )\mathrm {d}\xi$ converges to a finite number for $y\rightarrow \infty$. $\square$

The conditions (3.25) and (3.26) are somewhat implicit, so let us formulate more explicit conditions that imply them.

### Lemma 3.6

Assume that $\rho$, *v* and *z* exist, with $0<\rho <1$, $v<0$ and $z\ge f^{(2)}(y_c)$, such that

3.27 $$\begin{aligned} g(y)<v<0\quad \text {for}\quad y\ge z \end{aligned}$$

and

3.28 $$\begin{aligned} v\frac{\rho }{\Lambda }+\delta <0 \end{aligned}$$

with $\delta$ as introduced in $H_f$, then (3.25) holds.

### Proof

Let $\varepsilon >0$ be such that

$$\begin{aligned} v\frac{\rho }{\Lambda }+\delta +\varepsilon <0 \end{aligned}$$

Increasing *z* if necessary, we may assume that

$$\begin{aligned} f(y)<\delta +\varepsilon +y\quad \text {for}\quad y\ge z \end{aligned}$$

From (3.27) it follows that

$$\begin{aligned} \pi (\rho /\Lambda ,y)<y+v\frac{\rho }{\Lambda } \end{aligned}$$

Hence

$$\begin{aligned} f(\pi (\rho /\Lambda ,y))<\delta +\varepsilon +v\frac{\rho }{\Lambda }+y<y \end{aligned}$$

$$\square $$

### Corollary 3.7

If $g(y)=-wy$ we can satisfy (3.25) for any $\delta \ge 0$ in $H_f$ and any choice of $\rho \in (0,1)$.

For condition (3.26), the behaviour of *f* for $y\downarrow 0$ matters too. We therefore focus on

3.29 $$\begin{aligned} f(y)=y\left( 1+\frac{\sigma _2}{y}\right) ^{\sigma _1},\quad \text {with}\ \sigma _1>1,\ \sigma _2>0 \end{aligned}$$

which was derived in De Graaf et al. (2014).

### Lemma 3.8

Let *f* be given by (3.29).

i. If $g(y)=-wy$, condition (3.26) is fulfilled.
ii. If, for some $y_s>0$, $g(y)=-wy$ for $y\le y_s$ and
  3.30 $$\begin{aligned} \Lambda >w(\sigma _1-1) \end{aligned}$$
  condition (3.26) is fulfilled.

### Proof

For *f* given by (3.29) we have

$$\begin{aligned} f(y)=\sigma _2^{\sigma _1}y^{1-\sigma _1}(1+o(1))\quad \text {for}\quad y\downarrow 0 \end{aligned}$$

It follows that

$$\begin{aligned} f_-^{-1}(y)=\sigma _2^{\frac{\sigma _1}{\sigma _1-1}}y^{\frac{1}{1-\sigma _1}}(1+o(1))\quad \text {for}\quad y\rightarrow \infty \end{aligned}$$

For small enough *y* we have

$$\begin{aligned} T(y)=-\frac{1}{w}\log y+O(1) \end{aligned}$$

and consequently

$$\begin{aligned} \mathrm {e}^{-\Lambda T(f_-^{-1}(\xi ))}=\xi ^{\frac{\Lambda }{w(1-\sigma _1)}}O(1) \end{aligned}$$

If (3.30) holds, the exponent is less than $-1$ and boundedness of $\alpha$ suffices to fulfil (3.26). If $g(y)=-wy$ for all *y*, $\alpha (\xi )=\frac{\Lambda }{w\xi }(1+o(1))$ for $\xi \rightarrow \infty$ and the fact that the exponent $\frac{\Lambda }{w(1-\sigma _1)}$ is negative guarantees that (3.26) holds. $\square$

Before summarizing the results of this section, let us elucidate the role of the constant *c* in (3.20), (3.21). To compute the density of the fixed point of *K*, first solve (3.20) and (3.21) with $c=1$. When (3.25) and (3.26) hold, the integral

$$\begin{aligned} \int _{f(y_c)}^{\infty }\phi (\eta )\mathrm {d}\eta \end{aligned}$$

is well-defined. The renormalized function

3.31 $$\begin{aligned} \left( \int _{f(y_c)}^{\infty }\phi (\eta )\mathrm {d}\eta \right) ^{-1}\phi \end{aligned}$$

is the object of interest. The above analysis leads to the following conclusion:

### Theorem 3.9

Provided the conditions (3.25) and (3.26) are satisfied, the next-generation operator *K* has a unique fixed point. This fixed point has a density (3.31), where $\phi$ is the solution of (3.20), (3.21) corresponding to the choice $c=1$, or any other choice. Hence $\phi (y)>0$ for $f(y_c)<y<\infty$.

## The stationary distribution

Throughout this section we assume that the conditions (3.25) and (3.26) appearing in Lemma 3.5 are satisfied.

As demonstrated in Theorem 6.1 of Diekmann et al. (1998) (and likewise in Lemma’s 3.7 and 3.8 of Diekmann et al. (2003)), there is a one-to-one correspondence between fixed points of the next-generation operator and stationary distributions of the process itself. Here the relationship is rather simple, since the jump rate does not depend on the position in the state space (or, in another jargon, the risk of encountering the pathogen does not depend on the immune level). In fact we have

### Theorem 4.1

If *m* is a stationary distribution then *b* defined by

4.1 $$\begin{aligned} b(\Gamma )=m(f^{-1}(\Gamma )) \end{aligned}$$

is a fixed point of the next-generation operator and, conversely, if *b* is a fixed point of the next-generation operator then *m* defined by

4.2 $$\begin{aligned} m(\Gamma )=b(f(\Gamma )) \end{aligned}$$

is a stationary distribution.

The intuitive argument underlying Theorem 4.1 is that a steady distribution yields a steady stream of jumps with **uniform** departure rate $\Lambda$ and that $f^{-1}$ relates the point of arrival to the point of departure. So the probability per unit of time of “landing” in $\Gamma$ after a jump equals $\Lambda m(f^{-1}(\Gamma ))$ and, with *b* the fixed point of *K*, this equals $c b(\Gamma )$. Since both *b* and *m* are probability measures, *c* must be equal to $\Lambda$. For a formal proof see (Diekmann et al. 1998, 2003) and take the limit $t\downarrow 0$ in (6.10) of Diekmann et al. (1998) or (3.27) of Diekmann et al. (2003).

### Corollary 4.2

There exists a unique normalized stationary distribution. This distribution has a density and the density takes strictly positive values on $(0,\infty )$.

Our next aim is to establish the asymptotic stability of the stationary distribution by invoking results of Pichór and Rudnicki (2000). To do so, we need to make some preparations.

In the following we use the symbol *m* again to denote an arbitrary (so not necessarily a stationary) measure on $(0,\infty )$. We define

4.3 $$\begin{aligned} (Q_t\times m)(\Gamma )=\int _{(0,\infty )}Q_t(z,\Gamma )m(\mathrm {d}z) \end{aligned}$$

If *m* is a positive measure, so is $Q_t\times m$. In order to show that $Q_t\times m$ is a probability measure if *m* is a probability measure, we need the following observation.

### Lemma 4.3

$$Q_t(z,(0,\infty ))=1$$, $$\forall z\in (0,\infty )$$.

### Proof

We claim that

4.4 $$\begin{aligned} Q_t^k(z,(0,\infty ))=\frac{(\Lambda t)^k}{k!}\mathrm {e}^{-\Lambda t} \end{aligned}$$

Clearly (4.4) is true for $k=0$. Suppose (4.4) has been verified for $k=n$. Then

$$\begin{aligned} Q_t^{n+1}(z,(0,\infty ))= & {} \int _{[0,t)\times (0,\infty )} B_{\mathrm {d}s}^1(z,\mathrm {d}x)Q_{t-s}^n(x,(0,\infty )) \\= & {} \int _0^t\Lambda \mathrm {e}^{-\Lambda s}Q_{t-s}^n(f(\pi (s,z)),(0,\infty ))\mathrm {d}s\\= & {} \int _0^t\Lambda \mathrm {e}^{-\Lambda s}\frac{(\Lambda (t-s))^n}{n!}\mathrm {e}^{-\Lambda (t-s)}\mathrm {d}s=\Lambda \mathrm {e}^{-\Lambda t}\int _0^t\frac{(\Lambda \sigma )^n}{n!}\mathrm {d}\sigma \\= & {} \Lambda \mathrm {e}^{-\Lambda t}\frac{\Lambda ^nt^{n+1}}{(n+1)!}=\frac{(\Lambda t)^{n+1}}{(n+1)!}\mathrm {e}^{-\Lambda t} \end{aligned}$$

and the claim is verified. Finally,

$$\begin{aligned} Q_t(z,(0,\infty ))=\sum _{k=0}^{\infty }Q_t^k(z,(0,\infty ))=\mathrm {e}^{-\Lambda t}\sum _{k=0}^{\infty }\frac{(\Lambda t)^k}{k!}=1 \end{aligned}$$

$$\square $$

If *m* has a density, does $Q_t\times m$ have a density? Or, in other words, does the semigroup of operators associated with the kernel $Q_t$ leave the subspace of absolutely continuous measures invariant, so that we can associate with this kernel a semigroup of operators on $L_1(0,\infty )$? The answer is probably affirmative without severe conditions on *g* and *f*. Below we shall derive a stronger result under a condition that guarantees the monotonicity of

$$\begin{aligned} s\mapsto \pi (t-s,f(\pi (s,z))) \end{aligned}$$

so of the position at time *t* when starting in *z*, given that precisely one jump occurs, as a function of the time *s* at which the jump occurs. But first we pay attention to the situation with no jump at all.

### Lemma 4.4

If *m* has a density $\phi$, then $Q_t^0\times m$ has a density $T_0(t)\phi$ defined by

4.5 $$\begin{aligned} (T_0(t)\phi )(y)=\mathrm {e}^{-\Lambda t}\frac{g(\pi (-t,y))}{g(y)}\phi (\pi (-t,y)) \end{aligned}$$

### Proof

$$\begin{aligned} (Q_t^0\times m)(\Gamma )= & {} \int _{(0,\infty )}Q_t^0(z,\Gamma )\phi (z)\mathrm {d}z=\mathrm {e}^{-\Lambda t}\int _{(0,\infty )}\delta _{\pi (t,z)}(\Gamma )\phi (z)\mathrm {d}z\\= & {} \mathrm {e}^{-\Lambda t}\int _{\pi (-t,\Gamma )}\phi (z)\mathrm {d}z=\mathrm {e}^{-\Lambda t}\int _{\Gamma }\phi (\pi (-t,\eta ))\frac{g(\pi (-t,\eta ))}{g(\eta )}\mathrm {d}\eta \end{aligned}$$

where in the last step we used the identity (2.4). $\square$

The condition alluded to above reads

4.6 $$\begin{aligned} \frac{f'(y)g(y)}{g(f(y))}<1,\quad \forall y\in (0,\infty ) \end{aligned}$$

(We provide the biological interpretation of (4.6) after the next lemma.)

### Lemma 4.5

If (4.6) holds, the function

$$\begin{aligned} s\mapsto \pi (t-s,f(\pi (s,z))) \end{aligned}$$

is, for every given $z\in (0,\infty )$ and $t>0$, an increasing function on [0, *t*].

### Proof

$$\begin{aligned} \pi (t-s,f(\pi (s,z)))=T^{-1}(t-s+T(f(\pi (s,z)))) \end{aligned}$$

and $T^{-1}$ is decreasing, so it suffices to prove that

$$\begin{aligned} s\mapsto t-s+T(f(\pi (s,z))) \end{aligned}$$

is decreasing as well. The derivative with respect to *s* is

$$\begin{aligned} -1+\frac{1}{g(f(\pi (s,z)))}f'(\pi (s,z))g(\pi (s,z)) \end{aligned}$$

and (4.6) guarantees that this is negative. $\square$

The interpretation is that, by postponing the jump, you end up higher on the *y*-axis. And (4.6) is indeed an infinitesimal version of exactly this condition:

$$\begin{aligned} f(y)+g(f(y))\mathrm {d}t<f(y+g(y)\mathrm {d}t)=f(y)+f'(y)g(y)\mathrm {d}t+o(\mathrm {d}t) \end{aligned}$$

Our motivation for this assumption derives from

### Lemma 4.6

With

$$\begin{aligned} f(y)=y\left( 1+\frac{\sigma _2}{y}\right) ^{\sigma _1},\quad \sigma _2>0,\ \sigma _1>1, \end{aligned}$$

and

$$\begin{aligned} g(y)=-wy,\quad w>0, \end{aligned}$$

condition (4.6) holds.

### Proof

A straightforward calculation reveals that

$$\begin{aligned} f'(y)g(y)-g(f(y))=-w\left( 1+\frac{\sigma _2}{y}\right) ^{\sigma _1-1}(-\sigma _2\sigma _1)>0 \end{aligned}$$

$$\square $$

### Lemma 4.7

Assume that (4.6) holds. Denote the inverse function of

$$\begin{aligned} \eta =\eta (s)=\pi (t-s,f(\pi (s,y))) \end{aligned}$$

by $S=S(\eta )$ (where we have suppressed the dependence of *S* on *t* and *y* in the notation). Then

4.7 $$\begin{aligned} Q_t^1(y,\Gamma )=\Lambda \mathrm {e}^{-\Lambda t}\int _{\Gamma }q^1(t,y,\eta )\mathrm {d}\eta \end{aligned}$$

with

4.8 $$\begin{aligned} q^1(t,y,\eta ):=\mathbb {1}_{[\pi (t,f(y)),f(\pi (t,y))]}(\eta )\frac{\mathrm {d}S}{\mathrm {d}\eta }(\eta ) \end{aligned}$$

### Proof

$$\begin{aligned} Q_t^1(y,\Gamma )= & {} \int _{[0,t)\times (0,\infty )}\Lambda \mathrm {e}^{-\Lambda s}\delta _{f(\pi (s,y))}(\mathrm {d}x)\mathrm {e}^{-\Lambda (t-s)}\delta _{\pi (t-s,x)}(\Gamma )\mathrm {d}s\\= & {} \Lambda \mathrm {e}^{-\Lambda t}\int _0^t\delta _{\pi (t-s,f(\pi (s,y)))}(\Gamma )\mathrm {d}s=\Lambda \mathrm {e}^{-\Lambda t}\int _{\pi (t,f(y))}^{f(\pi (t,y))}\delta _{\eta }(\Gamma )\frac{\mathrm {d}S}{\mathrm {d}\eta }(\eta )\mathrm {d}\eta \\= & {} \Lambda \mathrm {e}^{-\Lambda t}\int _{[\pi (t,f(y)),f(\pi (t,y))]\cap \Gamma }\frac{\mathrm {d}S}{\mathrm {d}\eta }(\eta )\mathrm {d}\eta \\= & {} \Lambda \mathrm {e}^{-\Lambda t}\int _{\Gamma }\mathbb {1}_{[\pi (t,f(y)),f(\pi (t,y))]}(\eta )\frac{\mathrm {d}S}{\mathrm {d}\eta }(\eta )\mathrm {d}\eta \end{aligned}$$

$$\square $$

Note that $q^1(t,y,\eta )\ge 0$ and in fact is **strictly** positive for

$$\begin{aligned} \pi (t,f(y))<\eta <f(\pi (t,y)) \end{aligned}$$

which in the limit $t\rightarrow \infty$ amounts to $\eta \in (0,\infty )$.

### Corollary 4.8

Assume (4.6) holds. $Q_t^1\times m$ has density

$$\begin{aligned} y\mapsto \Lambda \mathrm {e}^{-\Lambda t}\int _{(0,\infty )}q^1(t,z,y)m(\mathrm {d}z) \end{aligned}$$

In particular, if *m* has a density $\phi$, then $Q_t^1\times m$ has density $T_1(t)\phi$ defined by

4.9 $$\begin{aligned} (T_1(t)\phi )(y)=\Lambda \mathrm {e}^{-\Lambda t}\int _{(0,\infty )}q^1(t,z,y)\phi (z)\mathrm {d}z \end{aligned}$$

(we say that $T_1(t):L_1\rightarrow L_1$ is a kernel operator).

### Lemma 4.9

If $Q_t^1(y,\Gamma )=\Lambda \mathrm {e}^{-\Lambda t}\int _{\Gamma }q^1(t,y,\eta )\mathrm {d}\eta$ then for $n\ge 1$

4.10 $$\begin{aligned} Q_t^n(y,\Gamma )=\frac{\Lambda ^n\mathrm {e}^{-\Lambda t}}{n!}\int _{\Gamma }q^n(t,y,\eta )\mathrm {d}\eta \end{aligned}$$

where $q^n$ is inductively defined by

$$\begin{aligned} q^{n+1}(t,y,\eta )=(n+1)\int _0^tq^n(t-s,f(\pi (s,y)),\eta )\mathrm {d}s \end{aligned}$$

### Proof

$$\begin{aligned} Q_t^{n+1}(y,\Gamma )= & {} \int _{[0,t)\times (0,\infty )}\Lambda \mathrm {e}^{-\Lambda s}\delta _{f(\pi (s,y))}(\mathrm {d}x)Q_{t-s}^n(x,\Gamma )\mathrm {d}s \\= & {} \int _0^t\Lambda \mathrm {e}^{-\Lambda s}Q_{t-s}^n(f(\pi (s,y)),\Gamma )\mathrm {d}s \end{aligned}$$

So if (4.10) holds, then

$$\begin{aligned} Q_t^{n+1}(y,\Gamma )= & {} \int _0^t\Lambda \mathrm {e}^{-\Lambda s}\frac{\Lambda ^n\mathrm {e}^{-\Lambda (t-s)}}{n!}\int _{\Gamma }q^n(t-s,f(\pi (s,y)),\eta )\mathrm {d}\eta \mathrm {d}s\\= & {} \frac{\Lambda ^{n+1}\mathrm {e}^{-\Lambda t}}{(n+1)!}\int _{\Gamma }(n+1)\int _0^tq^n(t-s,f(\pi (s,y)),\eta ) \mathrm {d}s\mathrm {d}\eta \\= & {} \frac{\Lambda ^{n+1}\mathrm {e}^{-\Lambda t}}{(n+1)!}\int _{\Gamma }q^{n+1}(t,y,\eta )\mathrm {d}\eta \end{aligned}$$

$$\square $$

By comparing (4.10) and (4.4) we deduce that

4.11 $$\begin{aligned} \int _{(0,\infty )}q^n(t,y,\eta )\mathrm {d}\eta =t^n \end{aligned}$$

### Corollary 4.10

Assume (4.6) holds. If *m* has a density $\phi$, then $Q_t\times m$ has a density, say $T(t)\phi$. Moreover, for each $\phi \in L_1(0,\infty )$ the map $t\mapsto T(t)\phi$ from ${\mathbb {R}}_{+}$ to $L_1(0,\infty )$ is continuous and $T(t)-T_0(t)$ (recall (4.5)) is a kernel operator.

### Proof

Since translation is continuous in $L_1$, we deduce from (4.5) that $||T_0(t)\phi -\phi ||\rightarrow 0$ as $t\downarrow 0$. Combining (4.10) and (4.11) we find that $||T(t)\phi -T_0(t)\phi ||\rightarrow 0$ as $t\downarrow 0$ (in fact the stronger result $||T(t)-T_0(t)||\rightarrow 0$ as $t\downarrow 0$ holds). Since $\{T(t)\}$ is a semigroup, continuity at $t=0$ implies continuity. $\square$

In the terminology of Pichór and Rudnicki (2000) $\{T(t)\}$ is a Markov semigroup. From Corollary 4.2 we know that $\{T(t)\}$ has a unique invariant density $\psi$ and that $\psi$ is positive. The fact that $T(t)\ge T_1(t)$ and $||T_1(t)||=\Lambda t\mathrm {e}^{-\Lambda t}>0$ for $t>0$ guarantees that $\{T(t)\}$ is partially integral as defined in Pichór and Rudnicki (2000). Thus we verified all assumptions of Theorem 2 in Pichór and Rudnicki (2000) (and in fact also of Theorem 2 in the preprint (Gerlach and Glück 2017)) and we obtain

### Theorem 4.11

Assume (4.6) holds and assume *m* has a density $\phi$. Let $T(t)\phi$ be the density of $Q_t\times m$. Then

4.12 $$\begin{aligned} \displaystyle \lim _{t\rightarrow \infty }||T(t)\phi -\psi ||_{L_1}=0 \end{aligned}$$

where $\psi$ is the density corresponding to the normalized stationary distribution.

But what happens if *m* does not have a density? The fact that the singular part of $Q_t\times m$ converges to zero guarantees that in that case $Q_t\times m$ converges to ${\bar{m}}$ in the total variation norm, where ${\bar{m}}$ is the unique normalized stationary distribution. Indeed, we can write

$$\begin{aligned} Q_{t+s}\times m=Q_t\times Q_s\times m=Q_t\times (m_s^{\text {ac}}+m_s^{\text {sing}}) \end{aligned}$$

where $m_s^{\text {ac}}$ is the absolutely continuous part of $Q_s\times m$ and $m_s^{\text {sing}}$ the singular part. Then $||m_s^{\text {sing}}||\le \mathrm {e}^{-\Lambda s}$ and hence $||Q_t\times m_s^{\text {sing}}||\le \mathrm {e}^{-\Lambda s}$. Moreover, $||Q_t\times m_s^{\text {ac}}-||m_s^{\text {ac}}||{\bar{m}}||\rightarrow 0$ for $t\rightarrow \infty$. Hence

$$\begin{aligned} ||Q_{t+s}\times m-{\bar{m}}||= & {} ||Q_t\times m_s^{\text {ac}}-||m_s^{\text {ac}}||{\bar{m}}+||m_s^{\text {ac}}||{\bar{m}}-{\bar{m}}+Q_t\times m_s^{\text {sing}}||\\\le & {} ||Q_t\times m_s^{\text {ac}}-||m_s^{\text {ac}}||{\bar{m}}||+(1-||m_s^{\text {ac}}||)+\mathrm {e}^{-\Lambda s} \end{aligned}$$

and by choosing *s* sufficiently large the last two terms can be made arbitrarily small, while the first term can be made arbitrarily small by choosing *t* sufficiently large.

## Discussion

On the basis of a given force of infection $\Lambda$, a given waning rate *g* and a given boosting map *f*, we derived a constructive description of how the distribution of immune status of an individual changes in time. Under the not-so-very-restrictive conditions (3.25), (3.26) and (4.6) we established that the distribution converges to a unique stationary distribution that has an (almost) everywhere positive density.

Our approach builds on a long tradition as exposed in the seminal work (Lasota and Mackey 1994) of Lasota and Mackey. See (Rudnicki and Tyran-Kamińska 2015; Mackey et al. 2013; Lasota et al. 1992; Banasiak et al. 2012; Hille et al. 2016; Pichór and Rudnicki 2016). Also see the recent (Gerlach and Glück 2017), which builds on (and simplifies) work of Greiner (1982) and the somewhat older work of Heijmans (1986a, b) who uses spectral methods.

It might be interesting to explore yet another approach. Define

5.1 $$\begin{aligned} S(y):=\text {expected time till arrival in } \, y_c \, \text {when starting from } \, y>y_c \end{aligned}$$

Then *S* should satisfy the equation

5.2 $$\begin{aligned} S(y)={\hat{\tau }}(y,y_c)\mathrm {e}^{-\Lambda {\hat{\tau }}(y,y_c)}+\int _0^{{\hat{\tau }}(y,y_c)}\Lambda \mathrm {e}^{-\sigma \Lambda }(\sigma +S(f(\pi (\sigma ,y))))\mathrm {d}\sigma \end{aligned}$$

The point is that one needs *S* in order to compute the expected time between two passages of $y_c$ (indeed, the time till the first jump after passing $y_c$ is exponentially distributed and the time of the jump determines from which $y>y_c$ the waning back towards $y_c$ starts). If one manages to prove that the expected time between two passages of $y_c$ is finite, a general result, viz. Theorem V.1.2, page 126, of Asmussen (1987) guarantees existence, uniqueness and asymptotic stability (with respect to the weak $*$ topology) of a stationary distribution.

So far we considered the force of infection $\Lambda$ as a parameter. We now formulate a consistency condition that $\Lambda$ should satisfy. It involves three new modeling ingredients:

$$\mu $$:
the probability distribution of immune status at birth
$${\mathcal {F}}(a)$$:
the probability to survive till at least age *a*
$$\xi (y)$$:
the contribution to the force of infection when becoming infected while having immune status *y*

In a stationary population the stable age distribution has density $\tilde{{\mathcal {F}}}$ given by

$$\begin{aligned} \tilde{{\mathcal {F}}}(a)=\frac{{\mathcal {F}}(a)}{\int _0^{\infty }{\mathcal {F}}(\alpha )\mathrm {d}\alpha } \end{aligned}$$

The parameter $\Lambda$ should satisfy

$$\begin{aligned} \Lambda =\Lambda \int _0^{\infty }\xi (y)\int _0^{\infty }\int _0^{\infty }\tilde{{\mathcal {F}}}(a)Q_a(y_b,\mathrm {d}y)\mu (\mathrm {d}y_b)\mathrm {d}a \end{aligned}$$

and, since we are interested in $\Lambda >0$,

$$\begin{aligned} 1=\int _0^{\infty }\xi (y)\int _0^{\infty }\int _0^{\infty }\tilde{{\mathcal {F}}}(a)Q_a(y_b,\mathrm {d}y)\mu (\mathrm {d}y_b)\mathrm {d}a \end{aligned}$$

where the dependence of the right hand side on $\Lambda$ is hidden in the notation, but derives from the fact that *Q* depends on $\Lambda$.

The modeling approach presented here provides a basis for refining sero-epidemiological methods. These methods exploit serum antibodies as biomarkers for infection and yield powerful tools for inferring the frequency of asymptomatic infections, those that cannot be observed directly (De Melker et al. 2006; Kretzschmar et al. 2010). Our work was motivated by questions arising in the epidemiology of pertussis, where serological data suggest that exposure to pertussis occurs much more frequently than documented in notification data of national surveillance systems. Whether this is due to underascertainment and underreporting, or to the fact that many infections are asymptomatic or mild and therefore not diagnosed, is unclear. To design vaccination strategies that protect young infants, it would be very useful to understand better how the boosting and waning of immunity in a population is reflected in serological surveys of the population and what the distribution of serological markers can tell us about the risk of exposure to the pathogen.

Current estimates of incidence of seroconversions only account for backward recurrence time of the most recent infection, ignoring any previous history of infections (Teunis et al. 2012). Carry-over from prior infections, leading to elevated baseline antibody levels, influence the current seroconversion, through the function *f*. The function *f* also determines whether infection causes a “small” or a “large” jump in antibody level, putatively corresponding to asymptomatic or symptomatic infection, respectively (De Graaf et al. 2014).

Thus, the relation between an infection-history dependent degree of protection against symptomatic infection and the incidence with which infection events occur may be exploited to better characterize transmission in the population, not only for pertussis but for any infectious pathogen with a measurable serum antibody response.

The statistical methods that have been developed to estimate the incidence of infection from serological data might be adapted and extended in order to incorporate a continuum immunity status as in the current paper. While thinking about the possibilities, one quickly realizes that our assumption of an age-independent force of infection is doubtful. And although incorporation of an age-specific force of infection in the formalism does not lead to great difficulties, such an extension of course leads to major complications when it comes to parametrization and identification.

## Figures and Tables

**Fig. 2:**
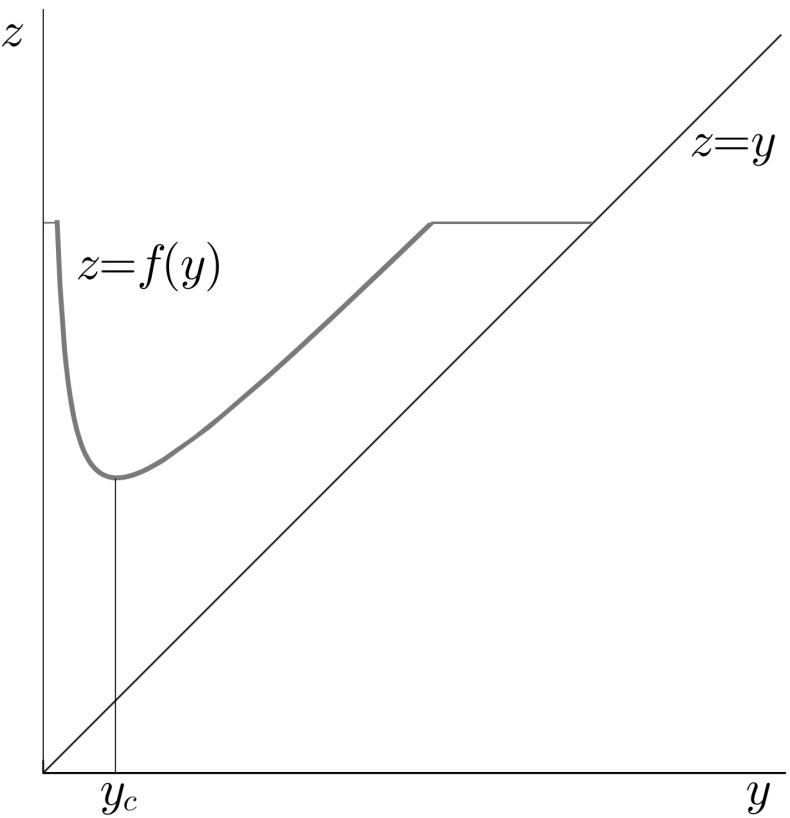
An alternative graph of *f*

## A1 Kolmogorov’s partial differential equations

The backward equation reads

A1.1 $$\begin{aligned} \frac{\partial u}{\partial t}(t,y)-g(y)\frac{\partial u}{\partial y}(t,y)=-\Lambda u(t,y)+\Lambda u(t,f(y)) \end{aligned}$$

while the forward equation is given by

A1.2 $$\begin{aligned} \frac{\partial m}{\partial t}(t,y)+\frac{\partial }{\partial y}(g(y)m(t,y))=-\Lambda m(t,y)+(Sm)(t,y) \end{aligned}$$

with *S* defined by

A1.3 $$\begin{aligned} (S\phi )(y)=\left\{ \begin{array}{ll} 0,&{} y<f(y_c)\\ \Lambda \phi (f_-^{-1}(y))\frac{1}{f'(f_-^{-1}(y))}+\Lambda \phi (f_+^{-1}(y))\frac{1}{f'(f_+^{-1}(y))},&{}y>f(y_c) \end{array} \right. \nonumber \\ \end{aligned}$$

Here $u(t,\cdot )$ is a continuous function of the form $u(t,\cdot )=\int _{(0,\infty )}Q_t(\cdot ,\mathrm {d}z)\psi (z)$, while $m(t,\cdot )$ is a density such that $\int _{\Gamma }m(t,y)\mathrm {d}y=\int _{(0,\infty )}\phi (z)Q_t(z,\Gamma )\mathrm {d}z$ for all $\Gamma \subset {(0,\infty )}$. One derives (A1.2) from (A1.1) by taking adjoints and restricting to absolutely continuous measures. This entails the assumption that one starts at $t=0$ with a density. Indeed, as the $Q_t^0(y,\Gamma )$ contribution to $Q_t(y,\Gamma )$ shows, the solution has a (diminishing) Dirac component when one starts with a Dirac initial condition.

## A2 An alternative type of function *f*

The motivation for a graph of *f* as depicted in Fig. 1 derives from the within-host submodel introducted in De Graaf et al. (2014). The behaviour for $y\downarrow 0$ and for $y\uparrow \infty$ is questionable. We conclude with a graphical representation, Fig. 2, of an alternative. Actually the proofs given above remain valid when $f(0)<\infty$ but the behaviour of *f*(*y*) for $y\rightarrow \infty$ is as described in $H_f$.

## Footnotes

1 Note that the two terms corresponding to $f_+^{-1}(y)$ as boundary of the integration interval annihilate each other.
